# Dinitroaniline Herbicide Resistance and Mechanisms in Weeds

**DOI:** 10.3389/fpls.2021.634018

**Published:** 2021-03-25

**Authors:** Jinyi Chen, Qin Yu, Eric Patterson, Chad Sayer, Stephen Powles

**Affiliations:** ^1^Australian Herbicide Resistance Initiative (AHRI), School of Agriculture and Environment, University of Western Australia (UWA), Perth, WA, Australia; ^2^Department of Plant, Soil, and Microbial Sciences, Michigan State University, East Lansing, MI, United States; ^3^Nufarm Limited, Melbourne, VIC, Australia

**Keywords:** dinitroaniline herbicides, trifluralin (herbicide), target-site resistance, tubulin mutations, non-target-site resistance, metabolic resistance

## Abstract

Dinitroanilines are microtubule inhibitors, targeting tubulin proteins in plants and protists. Dinitroaniline herbicides, such as trifluralin, pendimethalin and oryzalin, have been used as pre-emergence herbicides for weed control for decades. With widespread resistance to post-emergence herbicides in weeds, the use of pre-emergence herbicides such as dinitroanilines has increased, in part, due to relatively slow evolution of resistance in weeds to these herbicides. Target-site resistance (TSR) to dinitroaniline herbicides due to point mutations in α-tubulin genes has been confirmed in a few weedy plant species (e.g., *Eleusine indica*, *Setaria viridis*, and recently in *Lolium rigidum*). Of particular interest is the resistance mutation Arg-243-Met identified from dinitroaniline-resistant *L. rigidum* that causes helical growth when plants are homozygous for the mutation. The recessive nature of the TSR, plus possible fitness cost for some resistance mutations, likely slows resistance evolution. Furthermore, non-target-site resistance (NTSR) to dinitroanilines has been rarely reported and only confirmed in *Lolium rigidum* due to enhanced herbicide metabolism (metabolic resistance). A cytochrome P450 gene (CYP81A10) has been recently identified in *L. rigidum* that confers resistance to trifluralin. Moreover, TSR and NTSR have been shown to co-exist in the same weedy species, population, and plant. The implication of knowledge and information on TSR and NTSR in management of dinitroaniline resistance is discussed.

## Introduction

Weeds are a major threat to global food security. Weeds cause tremendous economic loss to agriculture by competing with crops for light, nutrition and water, and decreasing crop yield (Oerke, 2006). Herbicides are vital tools in controlling weeds, saving both time and labor, which in turn contributes immensely to global food production (Shaner, 2014). The first synthetic herbicide, 2,4-D, was produced in 1941 by Robert Pokorny and it is still being used today for broad-leaf weed control (Stephenson et al., 2001). After its discovery, new herbicide “modes of action” have been introduced approximately every 3 years, leading to the current availability of about 20 known modes of action.

However, persistent global herbicide application on large weed populations has resulted in the evolution of herbicide resistant weed populations. Since the first well documented herbicide resistant case for triazines in 1970 (Ryan, 1970), the total number of herbicide-resistant weed species has increased dramatically. A conservative estimate shows herbicide resistance has evolved in 262 weed species across 71 countries (Heap, 2021). What is worse, the number of herbicide resistant weed is still increasing, whereas development of new modes of action herbicides has been decreasing since 1991 (Duke, 2012).

With ever increasing herbicide resistance, understanding resistance mechanisms provides scientists and agronomists a theoretical framework to better control, mitigate, and manage herbicide resistant populations. This review focuses on the dinitroaniline herbicides and encompasses 1) the development of dinitroaniline herbicides, 2) resistance evolution and mechanisms, 3) inheritance, 4) potential fitness cost and 5) discussions on possible tactics to mitigate dinitroaniline resistance evolution.

## Development of Dinitroaniline Herbicides

**FIGURE 1:**
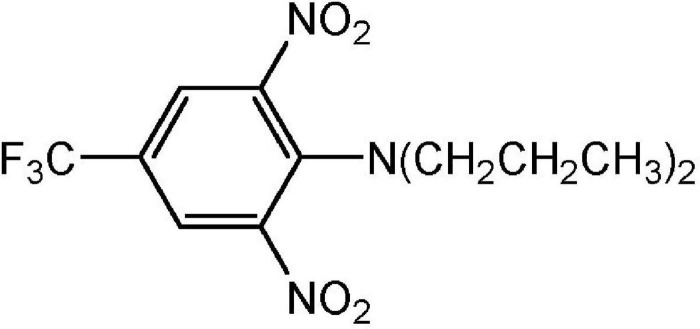
Trifluralin chemical structure.

Dinitroanilines represent a class of chemicals with a structure containing two nitro groups and an aromatic amine, aniline. Originally discovered in evaluations of dyes and dye chemical synthesis intermediates, dinitroanilines grew to be widely used in agriculture, industry and medical science (Dekker, 1999). In agriculture, dinitroanilines are mainly used as pre-emergence herbicides to control grass and some broadleaf weeds (Parka and Soper, 1977). The commercialized dinitroaniline herbicides so far include trifluralin, pendimethalin, ethalfluralin, oryzalin, butralin, benefin/benfluralin and prodiamine. The first dinitroaniline herbicide, trifluralin (α,α,α-trifluoro-2,6-dinitro-*N*,*N*-dipropyl-*p*-toluidine, Figure 1), was commercialized in the 1960s in the United States (Grover et al., 1997; Timmons, 2005). Originally it was used in soybean fields by pre-plant soil-incorporation for grass weed control (Epp et al., 2017). Later on, trifluralin was introduced into Latin America and Asia Pacific and extensively used in sugarcane and soybean in Brazil (Lima et al., 2018), and Australian cereal and legume fields (Jolley and Johnstone, 1994). With the introduction of newly developed, highly efficient post-emergence ALS- and ACCase-inhibitors in 1980s, trifluralin usage declined and the trifluralin market was significantly replaced by these newer herbicides. However, due to the rapid resistance evolution to these newer, post-emergence herbicides and the adoption of no-till or reduced tillage techniques for soil and moisture conservation, trifluralin has resurged in many markets. According to data from the Brazilian Institute of Environment and Renewable Natural Resources, sales of trifluralin comprised 1,887 tons in 2019 (Instituto Brasileiro do Meio Ambiente e dos Recursos Naturais Renováveis (IBAMA), 2020). In the United States, trifluralin was among the 25 most used pesticides in agriculture, and the estimated usage ranged from 1361 to 3175 tons in 2012 [Environmental Protection Agency (EPA), 2021]. In recent years trifluralin and pendimethalin have been the two most significant dinitroaniline herbicides used, estimated to represent a global farm gate value (the dollar amount of sales of product made to the actual farmer) of $USD 525 million. When considering trifluralin/pendimethalin, over 30% of the farm gate value is within the Australian and North American markets, and 50% of farm gate value is in cereals, cotton, vegetables and soybean. Controlling weed populations resistant to other herbicide chemistries is generally accepted as a significant factor driving dinitroaniline herbicide use in these regions and crops.

## Behavior and Mode of Action of Dinitroaniline Herbicides

Dinitroanilines have low water solubility (20°C in water, 0.22 mg/L for trifluralin, and 0.33 mg/L for pendimethalin). According to Environmental Protection Agency (EPA) 1996, trifluralin ranks as moderate to highly toxic for aquatic animals (fish and invertebrates) [Environmental Protection Agency (EPA), 1996]. Most dinitroaniline herbicides are highly volatile. At 25°C, the volatility of trifluralin is 9500 kPa, and pendimethalin, 1940 kPa (versus 3.17 kPa for water) (Congreve and Cameron, 2014). Dinitroanilines are subject to decomposition due to photodegradation (Wright and Warren, 1965), and its effectiveness is greatly affected by its soil incorporation depth (Savage and Barrentine, 1969; Spencer and Cliath, 1974). Therefore, dinitroaniline herbicides need to be incorporated with surface soil to minimize volatilization and photodegradation loss. Particularly, in Australia, the “incorporate by sowing” technique uses a knife point seeder to throw soil into the inter-row to cover the herbicide and reduce loss due to volatilization (Ashworth et al., 2010). When within the soil, dinitroanilines have strong binding coefficient (K_oc_, if K_oc_ > 4,000, non-mobile) with the soil (K_oc_ pendimethalin = 17,581, K_oc_ trifluralin = 15,800) (Helling, 1971, 1976; Congreve and Cameron, 2014), and slow microbial degradation. Microbial degradation is the primary breakdown route; however, persistence is generally long, often resulting in rotational crop limitations (Congreve and Cameron, 2014).

Due to low solubility in soil moisture and strong soil-binding, dinitroanilines enter the germinating seedlings primarily *via* gaseous absorption through the root, coleoptile node or hypocotyl, upon contact with the herbicide (Congreve and Cameron, 2014). Trifluralin soil-borne vapor plays an important phytotoxic role especially to plant roots (Barrentine and Warren, 1971; Charles and Richard, 1972). In early research on dinitroaniline herbicides uptake and translocation, it was found that in both monocots and dicots, the translocation of ^14^C-profluralin radioactivity to plant tops was very limited, while ^14^C-dinitramine was more readily translocated throughout the plant, and higher temperature (38°C) could help enhance the translocation to leaves (Hawxby, 1974). Limited trifluralin translocation to the aerial portions of the plant was reported in soybean and cotton plants (Strang and Rogers, 1971), and in *Alopecurus aequalis* seedlings (Hashim et al., 2012).

**FIGURE 2:**
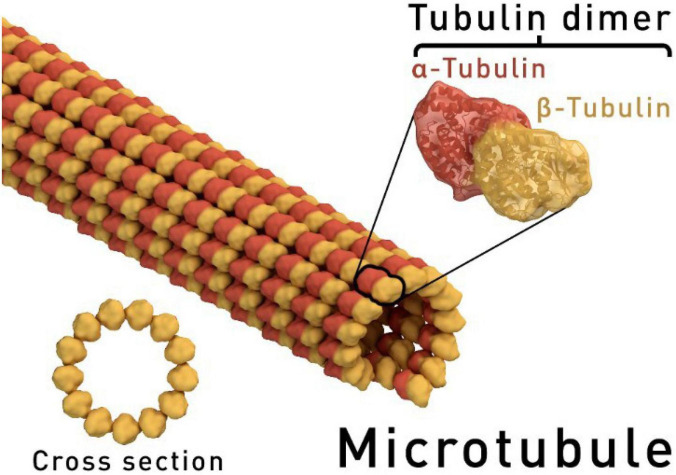
Simulative structure of a microtubule. The ring shape depicts a microtubule in cross-section, showing the 13 protofilaments surrounding a hollow centre (https://goo.gl/images/BKJ9m3, under the Creative Commons Attribution-Share Alike 4.0 International license).

Dinitroanilines target microtubules, which, together with microfilaments and intermediate filaments, are major components of the cytoskeleton. Microtubules are hollow cylinders, about 25 nm in diameter, that are comprised of α- and β-tubulin heterodimers (usually 13 protofilaments in eukaryotic cells) (Figure 2; Tilney et al., 1973; Desai and Mitchison, 1997; Downing and Nogales, 1998a, b). The α- and β-tubulins, each with a molecular weight of 50,000 daltons, share 36–42% amino acid sequence identity (Little and Seehaus, 1988). Microtubules perform various functions at different stages of cellular activity. During interphase, microtubules are critical for orchestrating cell wall synthesis in plant cells. Also, microtubules are anchored to the plasma membrane, forming cortical microtubules to help support cell shape. During mitosis, the bipolar spindle apparatus is comprised of microtubules and is capable of correctly positioning chromosomes to the cell midplane and then guiding separated chromatids to opposite ends of the cell (Shaw and Vineyard, 2014). To realize their mobile function(s), microtubules are required to be in a dynamic balance. There is a positive (+) and negative (−) end for microtubules; the positive end assembles the heterodimers using GTP, while the negative end dissociates into heterodimers. With balanced polymerization and de-polymerization, mitosis proceeds normally.

Dinitroanilines disrupt microtubule function by binding with unpolymerized tubulin heterodimers. *In vitro* analyses of *Chlamydomonas reinhardtii* tubulin showed specific binding with trifluralin, indicating that tubulin is the primary subcellular target of dinitroaniline action (Stokkermans et al., 1996). Similar for tubulin from *Zea mays* L., dinitroanilines bind to the unliganded α/β-tubulin heterodimers to form a herbicide-tubulin complex, and, with addition of the complex to the positive end of the growing microtubule, further elongation of the microtubule ceases (Hugdahl and Morejohn, 1993). Concomitantly, due to depolymerization of microtubules from the negative end, the microtubules become progressively shorter, eventually leading to their complete dissociation (Cleary and Hardham, 1988). When this occurs, mitosis is disturbed and mitotic cells are arrested in telophase. This is also supported by the cytological studies showing arrested mitosis at prometaphase due to loss of spindle microtubules, and formation of isodiametric cells in the elongation zone due to loss of cortical microtubules after trifluralin treatment (Vaughn, 1986). The cessation of cell division causes treated seedlings to exhibit swollen and stunted root symptoms (Lignowski and Scott, 1971). The affected seedlings either cannot emerge from the soil or there is no growth after emergence.

Interestingly, dinitroaniline herbicides target microtubules from both plants and protists, but not from animals or fungi (Chan and Fong, 1994; Bell, 1998; Dempsey et al., 2013), likely due to differences in binding affinities for animal tubulins. Oryzalin was found to bind *in vitro* to tubulins isolated from maize and *Chlamydomonas eugametos*, but not to purified tubulins from bovine brain tissue (Strachan and Hess, 1983; Morejohn et al., 1987; Hugdahl and Morejohn, 1993). Carrot is a notable exception and shows natural tolerance to dinitroaniline herbicides. Immunofluorescence and electron microscopy indicated that the microtubules of carrot roots were unaffected by dinitroaniline treatment (Vaughan and Vaughn, 1988).

Dinitroaniline herbicides inhibit the plant tubulin protein family, which is encoded by a multi-gene family comprised of several α- and β-tubulin genes. In the model plant *Arabidopsis thaliana*, there are at least six expressed α-tubulin genes and nine expressed β-tubulin genes (Snustad et al., 1992; Kopczak et al., 1992). In rice, there are three α-tubulin transcripts and eight β-tubulin genes reported (Qin et al., 1997; Yoshikawa et al., 2003). Similarly in *E. indica*, four β-tubulin and three α-tubulin isoforms have been identified (Waldin et al., 1992; Yamamoto et al., 1998; Yamamoto and Baird, 1999), whereas in *Setaria viridis*, two α- and two β-tubulin genes have been isolated (Waldin et al., 1992; Délye et al., 2004). In cross-pollinated *L. rigidum*, there are at least four α-tubulin isoforms, TUA1, TUA2, TUA3, and TUA4, but the number of transcripts coding for each isoform varies among individual/population (Chen et al., 2020b).

Crop selectivity of dinitroaniline herbicides is possible using physical herbicide positioning between crop and weed seeds in the soil. Crop seeds are beneath the layer of soil containing the dinitroaniline herbicide while the smaller annual weed seeds germinate on or near the soil surface. Furthermore, the trifluralin molecule has some species-specific selectivity and is generally more toxic to grass species (wheat, oats, barley, and rice) than broadleaf species (cotton, soybean, pea, and cucumber) (Barrentine and Warren, 1971). As trifluralin can be trapped in lipids, it is hypothesized that selective phytotoxicity of trifluralin in young seedlings is determined, in part, by the amount of endogenous lipids available to trap trifluralin and keep it from its site of phytotoxic action (Hilton and Christiansen, 1972).

## Resistance Evolution to Dinitroaniline Herbicides

Dinitroaniline herbicides have been used for more than 50 years; however, limited cases have been documented for dinitroaniline herbicide resistance in weedy species. Globally, thus far, populations of only seven weed species have been identified to be resistant to dinitroaniline herbicides (Heap, 2021): *E. indica*, *S. viridis*, *Amaranthus palmeri*, *Alopecurus myosuroides*, *Lolium rigidum*, *Poa annua* and *Alopecurus aequalis*. These resistant weed populations are mostly in Australia, America, and Japan, where dinitroaniline herbicides have been intensively used.

*E. indica* from South Carolina was among the first reports of dinitroaniline resistance following about a decade’s persistent application (Mudge et al., 1984). Subsequently, two more dinitroaniline-resistant biotypes of *E. indica* (i.e., resistant and intermediate resistant) were characterized (Vaughn et al., 1990). Later resistance was found in populations of *A. palmeri* and *P. annua* from other parts of the Carolinas and Georgia (Gossett et al., 1992; Lowe et al., 2001; Isgrigg et al., 2002).

*Setaria viridis* is the major dinitroaniline-resistant weed species reported in Canada. Trifluralin-resistant *S. viridis* populations were first found in southern and southwestern Manitoba (Morrison et al., 1989), and the resistance persisted for at least 7 years (Andrews and Morrison, 1997). In United Kingdom, one chlorotoluron-resistant *A. myosuroides* population was reported to metabolize and thus resist pendimethalin but not trifluralin (James et al., 1995). In Japan, trifluralin-resistant *A. aequalis* populations were identified after more than two decades of dinitroaniline usage (Hashim et al., 2012).

*Lolium rigidum* from Australia is prone to herbicide resistance evolution, and dinitroaniline herbicides are no exception (McAlister et al., 1995). Periodic herbicide resistance surveys in Australian agricultural areas show that more than 50% surveyed populations from South Australia and Victoria, and 26% from the Western Australia have become trifluralin resistant (Boutsalis et al., 2006; Owen et al., 2007, 2014). Among others, this is likely ascribed to reduced herbicide control efficacy due to high *L. rigidum* seed numbers, low herbicide doses (caused by dry environments, microbial metabolism of the herbicides etc.), and perhaps more importantly, development of metabolism-based cross-resistance in *L. rigidum* populations selected by other herbicides (e.g., Han et al., 2020). With increasing usage of trifluralin, more trifluralin-resistant *L. rigidum* populations are expected.

Relatively slow evolution of dinitroaniline herbicide resistance in weeds is likely due to several reasons: (1) Plants surviving the pre-emergence herbicide treatment early in the season can still be controlled by the application of herbicides from different modes of action, applied post-emergent, as well as by any other control methods implemented; (2) Use mixtures of pre-emergence herbicides with different modes of action significantly improves weed control efficacy (Soltani et al., 2020); and (3) Other factors like the existence of multiple target isoforms, genetic control mechanisms, and fitness costs associated with resistance alleles, as discussed below.

## Resistance Mechanisms to Dinitroaniline Herbicides

Generally, herbicide resistance mechanisms can be divided into target-site resistance (TSR) and non-target-site resistance (NTSR). TSR refers to resistance caused by the changes in herbicide target protein including mutation, duplication and overexpression, while NTSR includes all resistance mechanisms bypassing the TSR, primarily anything that reduces the amount of herbicide reaching the target protein such as alterations in absorption, translocation or metabolism (Powles and Yu, 2010). TSR is relatively easy and straightforward to study when the target protein is not part of a multi gene family and in diploid plant species, whereas unraveling NTSR mechanisms is more technically challenging and requires a more in depth understanding of the weed’s genetics and physiology.

### TSR Mechanisms to Dinitroaniline Herbicides

Dinitroanilines mainly target α- and β-tubulin in protists and higher plants. Resistance mutations identified in protists offer valuable reference. In protists, the first α-tubulin mutation (Tyr-24-His) conferring resistance to dinitroanilines was identified in a single-celled algae: *Chlamydomonas reinhardtii* (James et al., 1993). Later, more resistance mutations were documented in *Toxoplasma gondii* (Morrissette et al., 2004; Ma et al., 2007, 2008; Lyons-Abbott et al., 2010) and *Tetrahymena thermophila* (Lyons-Abbott et al., 2010). In weedy plants, the first resistance-endowing tubulin mutation (Thr-239-Ile) was characterized in *E. indica* (Anthony et al., 1998; Yamamoto et al., 1998). Subsequently, the same mutation was also identified in the protist *T. gondi* (Morrissette et al., 2004) and in weedy species *S. viridis* (Délye et al., 2004) and more recently in *L. rigidum* (Chen et al., 2018b). There are other resistance mutations identified in other plants (Table 1) and some of them are shared by plants and protists (Table 2). Thus far, there is a greater number of resistance mutations identified in protists than in plants (Morrissette et al., 2004; Pham and Morrissette, 2019), which may be due to their shorter life cycles, simple genome composition and laboratory-based forced selection/evolution in protists. Given the equal sensitivity to dinitroaniline herbicides in protists and in plants, more resistance-endowing mutations discovered in protists are expected to be eventually detected in plants, as dinitroaniline selection pressure continues and/or increases.

**TABLE 1:** Mutations in α- and β-tubulin identified in field-evolved dinitroaniline-resistant plants.

Mutation Site	Organism	Wild type amino acid	Substituted amino acid	References
**α-tubulin**				
125	*A. aequalis*	Leu	Met	Hashim et al., 2012
136	*A. aequalis S. viridis*	Leu	Phe	Délye et al., 2004
202	*A. aequalis L. rigidum*	Val	Phe	Hashim et al., 2012; Fleet et al., 2017; Chen et al., 2018b
239	*E. indica S. viridis L. rigidum*	Thr	Ile	Anthony et al., 1998; Délye et al., 2004; Yamamoto et al., 1998; Fleet et al., 2017; Chen et al., 2018b
243	*L. rigidum*	Arg	Met/Lys	Chu et al., 2018
268	*E. indica*	Met	Thr	Yamamoto et al., 1998
390 + 442*	*L. rigidum*	Arg, Asp	Cys, Glu	Chen et al., 2020b
**β-tubulin**				
241	*Poa annual L.*	Arg	Lys	Lowe et al., 2001

*390 + 442 double mutation confers resistance to dinitroaniline herbicides.

**TABLE 2:** Common α-tubulin mutations and mutation sites shared by the protozoan *Toxoplasma* and higher plants.

Site	Amino acid substitution	Tubulin Gene (Isoform)	Organism	References
136	Leu136Phe	AAA21350.1 (α-tubulin)	*T. thermophila*	Lyons-Abbott et al., 2010
	Leu136Phe	XP_002364807.1 (α1-tubulin)	*T. gondii*	Morrissette et al., 2004
	**Leu136Phe**	**BAJ06363.1 (TUA1)**	***A. aequalis***	**Hashim et al., 2012**
	**Leu136Phe**	**CAE52515.1 (α2-tubulin)**	***S. viridis***	**Délye et al., 2004**
239	Thr239Ile	XP_002364807.1 (α1-tubulin)	*T. gondii*	Morrissette et al., 2004
	**Thr239Ile**	**AAC05717.1 (TUBA1)**	***E. indica***	Yamamoto et al., 1998; Anthony and Hussey, 1999
	**Thr239Ile**	**MT514937 (TUA4)**	***L. rigidum***	**Fleet et al., 2017; Chen et al., 2018b**
	**Thr239Ile**	**CAE52515.1 (α2-tubulin)**	***S. viridis***	**Délye et al., 2004**
268	Met268Thr	XP_002364807.1 (α1-tubulin)	*T. gondii*	Ma et al., 2007
	**Met268Thr**	**AAC05717.1 (TUBA1)**	***E. indica***	**Yamamoto et al., 1998**
243	Arg243Cys	XP_002364807.1 (α1-tubulin)	*T. gondii*	Morrissette et al., 2004
	Arg243Ser	XP_002364807.1 (α1-tubulin)	*T. gondii*	Morrissette et al., 2004
	**Arg243Lys**	**MT514937 (TUA4)**	***L. rigidum***	**Chu et al., 2018**
	**Arg243Met**	**MT514937 (TUA4)**	***L. rigidum***	**Chu et al., 2018**

*Information about plant species is in bold.*

Target-site resistance in the obligatory cross-pollinated *L. rigidum* is more complicated when compared with self-pollinated weed species, mostly due to *Lolium* genetic diversity. At least four α-tubulin isoforms (named TUA1–TUA4) have been identified in plants from two *L. rigidum* populations, with resistance-endowing mutations occurring largely in TUA3 and TUA4, according to our sequencing results in several resistant populations (Chen et al., 2018b, 2020b; Chu et al., 2018). Moreover, the same α-tubulin isoform from a single plant can be encoded by transcripts with differences only in untranslated regions (UTR), and thus adding another layer of complexity to tubulin gene sequencing and cloning (Chen et al., 2020b). In addition, there exists a substantial amount of synonymous mutation among α-tubulin transcripts encoding the same isoform from different populations, which challenges the success of PCR amplification by using a single primer pair (Chen et al., 2020b). This discovery suggests care should be taken when attempting to definitively determine the presence or absence of TSR mutations if the herbicide target is derived from a multiple gene family, even in a diploid species (i.e., it is similar to herbicides that target single genes in polypoid species). Also, it is worth noting that, due to genetic variation in different populations or even individuals within a population, one cannot entirely rely on RNA-seq information collected from a small number of plants. In these cases, validation of RNA-seq data by isoform-specific PCR is critical, as errors in alignment and assembly can occur when assembling highly similar sequences from the same gene family.

Due to the redundancy of function in a multi-gene family like tubulin, a greater level of diversity in resistance mutations is possible at both the population and individual level. For instance, at least three TUA4 mutations were previously identified in *L. rigidum* population M4/16, including Val-202-Phe, Thr-239-Ile, and Arg-243-Lys/Met (Chen et al., 2018b; Chu et al., 2018), and individuals with different combinations of mutations (e.g., 202 + 243, 202 + 239, or 239 + 243) have also been identified (Fleet et al., 2017; Chu et al., 2018) most likely due to obligate out-crossing in *Lolium*. More than two resistance alleles can exist in a single plant due to presence of multiple gene copies or transcripts. For instance, a plant homozygous for the Val-202-Phe mutant alleles in TUA4 also has the Arg-390-Cys + Asp-442-Glu resistance allele in TUA3 (Chen et al., 2020b). Nevertheless, the frequency of resistance mutations varies even among individuals of a population. In one study, 39 resistant *L. rigidum* plants were analyzed. The Val-202-Phe mutation was most commonly found (90%), with the Arg-243-Met mutation occurring in only 7% of screened plants. Furthermore, no plants were identified as homozygous for the Arg-243-Met mutation (Chen et al., 2018b; Chu et al., 2018). This is likely related to the herbicide selection pressure and fitness penalties associated with this mutant allele (see below). As more resistant populations/plants are analyzed, a clearer picture of resistance mutation frequency will be determined in *L. rigidum*, which may be echoed in other plant species.

Unlike TSR to other herbicides (e.g., glyphosate) involving target gene duplication/overexpression, the chance is rare for evolution of such TSR mechanisms to dinitroaniline herbicides in weeds, unless both α- and β-tubulin are involved. This is expected, as the amount of α- and β-tubulin isoforms are in dynamic balance for cell vitality, and overexpression of either of the two may be lethal (Anthony and Hussey, 1998). In addition, tubulins are structural proteins and constitutively expressed in abundance, thus overexpression is a less-likely mechanism for conferring resistance.

### NTSR Mechanisms to Dinitroaniline Herbicides

No difference in herbicide uptake and/or translocation has so far been ascribed to dinitroaniline resistance (McAlister et al., 1995; Hashim et al., 2012), although dinitroaniline translocation patterns are herbicide-, weed species-, and experiment-dependent (Hawxby, 1974). This is in line with the fact that dinitroaniline herbicides are often phytotoxic to germinating seedlings and therefore little whole plant translocation of the herbicide is needed for activity. Instead, thus far, enhanced dinitroaniline metabolism (metabolic resistance) has been demonstrated as the main NTSR mechanism in studied weed species. It has been challenging to identify the major trifluralin metabolic pathway and to isolate trifluralin metabolites in plants (Probst et al., 1967; Biswas and Hamilton, 1969), and extraction and quantification of dinitroaniline metabolites is hindered by the highly volatile nature of dinitroaniline herbicides. These difficulties have meant that TSR has been the primary research focus in understanding resistance mechanisms to dinitroanilines in plants. Nevertheless, essential dinitroaniline herbicide metabolites are recently identified using yeast-expressed plant cytochrome P450 enzymes (see below) (Abdollahi et al., 2021).

There is some indirect evidence for enhanced dinitroaniline herbicide metabolism in resistant weed species. In *A. myosuroides*, a single population that can metabolize chlorotoluron (and that is cross-resistant to pendimethalin) is thought to be metabolically resistant due to oxidative degradation of the 4-methyl group in pendimethalin (James et al., 1995). In *L. rigidum*, the P450 inhibitor malathion showed a synergistic effect with pendimethalin (Tardif and Powles, 1999), and phorate [another cytochrome P450 inhibitor (Ferhatoglu et al., 2005)] can partially reverse trifluralin resistance (Busi et al., 2017). Recently, enhanced trifluralin metabolism in several *L. rigidum* populations has been identified and a diagnostic assay using ^14^C-trifluralin established for metabolic resistance (Chen et al., 2018a). Furthermore, a cytochrome P450 gene, CYP81A10v7, has been identified and characterized from a trifluralin resistant *L. rigidum* population. When rice seedlings were transformed with a CYP81A10v7 over-expression construct, they became moderately resistant to trifluralin (Han et al., 2020). This is the first metabolic gene identified that is clearly associated with the evolution of trifluralin resistance. It should be noted that the resistance level in the transgenic rice is lower than what was observed in the resistant *L. rigidum* plants. Recently, the CYP706 family from Arabidopsis and other plant species have been demonstrated to be able to metabolize most dinitroaniline herbicides (essentially to mono- and di-oxygenated compounds), including trifluralin, pendimethalin and ethalfluralin, and thus weeds have potential for the evolution of dinitroaniline metabolic resistance if these P450 genes are selected (Abdollahi et al., 2021). Taken together, these studies affirm that metabolism is a viable NTSR mechanism to dinitroanilines in weeds, though much remains to be revealed.

It is common for weeds to evolve both TSR and NTSR to commonly used herbicides and, especially in cross-pollinated species, for those mechanisms to be stacked in the same population or in the same plants. This has been previously shown for other high-use herbicides such as glyphosate, ALS- or ACCase-inhibitors (Délye, 2013; Yu and Powles, 2014; Duke, 2019), and now in dinitroanilines. For instance, in a *L. rigidum* population (202FT), both target-site mutations and non-target-site herbicide metabolism contribute to trifluralin resistance (Chen et al., 2018a, b, 2020a, b). The prevalence of each mechanism in various populations leads us to assume that more populations containing both TSR and NTSR are to be uncovered, at least for *L. rigidum*.

## Genetic Inheritance of Dinitroaniline Resistance

Genetic inheritance studies of dinitroaniline resistance have been carried out in two self-pollinated resistant weed species: *E. indica* (Zeng and Baird, 1997) and *S. viridis* (Jasieniuk et al., 1994). In both cases, resistance was conferred by TSR, later revealed as the α-tubulin mutations Thr-239-Ile and Met-268-Thr in *E. indica* (Anthony et al., 1998; Yamamoto et al., 1998), and Leu-136-Phe and Thr-239-Ile in *S. viridis* (Délye et al., 2004). Interestingly, and contrary to most heredity patterns of TSR, TSR to dinitroaniline herbicides in these weed species were reported to be recessive traits.

A similar inheritance study for dinitroanilines was recently carried out in one *L. rigidum* population (202FT) (Chen et al., 2019). Plants of this population are homozygous for the Val-202-Phe mutation in TUA4. Generally, dominance of the resistance trait and gene loci contribution to herbicide resistance are rate-dependent. It was shown that at 480 g ha^–1^ trifluralin (half of the field rate), resistance is inherited as a single, recessive nuclear gene trait, similar to what has been shown in *E. indica* (Zeng and Baird, 1997) and *S. viridis* (Délye et al., 2004). However, at the lower rate of 120 g. ha^–1^ trifluralin, the resistance trait does not follow a single gene, recessive pattern, indicating other unknown but possibly weak TSR or even metabolism-based NTSR mechanisms involved in resistance.

Given the recessive nature of TSR to dinitroaniline resistance at the field relevant rates, and dinitroanilines targeting a small nuclear gene family with multiple gene copies, it follows that dinitroaniline resistant plants should be rare. However, dinitroaniline herbicide resistance evolution in weeds should not be underestimated, especially in cross-pollinating weed species with high levels of genetic diversity. Cross-pollinated species (e.g., *L. rigidum*) are capable of accumulating numerous resistance-conferring genes/mutations, either TSR or NTSR, in single plants quickly due to the sheer number of plants and their obligate cross-pollination. Another factor that may increase rates of dinitroaniline resistance evolution is that lower-than-field-rate levels of dinitroaniline are often encountered when environmental and soil conditions are unfavorable during or shortly after herbicide application. In these situations, minor resistance genes from the standing variations can be enriched and selected for, as has been demonstrated for resistance evolution to other pre-emergence herbicides under recurrent low herbicide rate selection (Busi and Powles, 2016).

## Fitness Cost of Dinitroaniline Resistance

So far, limited fitness studies on dinitroaniline herbicide resistance have been conducted. In protists, like the haploid parasite *T. gondii*, various α-tubulin mutations (at positions 136, 239, 243 and 268) confer dinitroaniline resistance at a cost to microtubule function (Ma et al., 2008). Interestingly, when α-tubulin mutants (Phe-52-Tyr) were grown without dinitroanilines, they spontaneously acquired a secondary mutation (Ala-273-Val or Asp-367-Val) which increased parasite fitness, although resistance level of mutants with the double mutation also decreased (Ma et al., 2007). Similarly, more secondary mutations were detected in parasites including the Phe-52-Tyr or Glu-142-Ser α-tubulin mutations, which reduced resistance but helped improve fitness (Ma et al., 2008). It remains to be determined if the compensating tubulin mutations also occur in other species, including higher plants.

**FIGURE 3:**
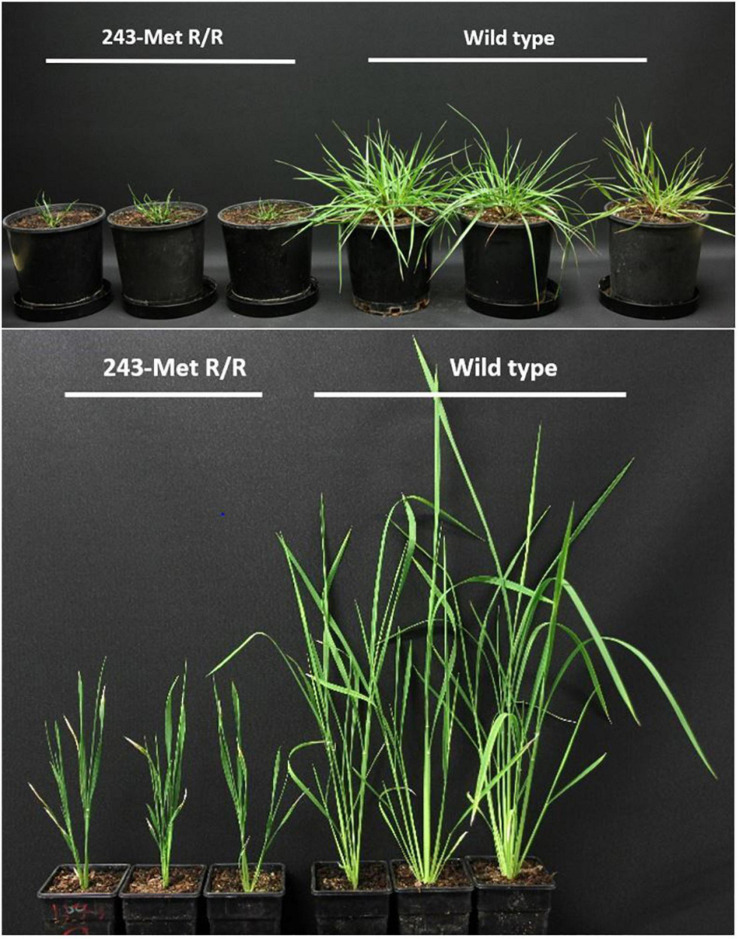
Helical growth of *Lolium rigidum* (top picture, on the left) and transgenic rice (T2, bottom picture, on the left) homozygous for the α-tubulin Arg-243-Met mutation (243-Met R/R) in comparsion to the norma growth of corresponding wild type plants (on the right). Photos were taken 42 and 30 days after *L. rigidum* and rice transplanting, respectively.

In higher plants, a fitness cost study using near-isogenic *Setaria* lines conducted in the greenhouse and in the field showed that, without herbicide treatment, plants homozygous for the Thr-239-Ile mutation were smaller and had lower 1,000-grain weight (Darmency et al., 2011). In *L. rigidum*, potential fitness cost on plant biomass was observed in plants homozygous for the Arg-243-Met mutation (Chu et al., 2018). Notably, the frequency of the Arg-243-Met mutation in the field-collected *L. rigidum* population (M4/16) was found to be low. Only two 243-Met heterozygotes were uncovered in 39 resistant plants analyzed, while homozygous resistant plants were not detected. This disequilibrium may result from a severe fitness cost of this mutation. Controlled greenhouse crosses of Arg-243-Met *L. rigidum* heterozygotes produced Arg-243-Met homozygotes exhibiting severe dwarfism and right-handed helical growth (Figure 3). Furthermore, homozygous rice plants transformed with the *Lolium* Arg-243-Met mutant tubulin gene also exhibited dwarf and helical growth (Figure 3), indicating that this Arg-243-Met α-tubulin mutation confers aberrant plant morphology, although the cellular basis of these abnormalities remains to be investigated.

Preliminary observation of *L. rigidum* indicates a severe cost to TSR based dinitroaniline resistance in plant vegetative growth (Chu et al., 2018). In both *L. rigidum* and transgenic rice plants heterozygous for the 243-Met mutant allele, no altered growth phenotypes have been observed. This suggests that both the cost of resistance and the resistance itself are recessive. This is similar to the fitness cost associated with the target-site mutations (e.g., Asp-2078-Gly mutation) to ACCase-inhibiting herbicides (Menchari et al., 2008; Vila-Aiub et al., 2015) and TIPS to glyphosate (Han et al., 2017). These growth abnormalities associated with the homologous 243-Met mutant allele starkly contrast with the lack of visible growth defects shown for the most commonly identified α-tubulin mutation, Val-202-Phe in *L. rigidum*, similar to the most popular ACCase resistance mutation: Ile-1781-Leu (Wang et al., 2010). This may explain the much higher frequency of the Val-202-Phe than the Arg-243-Met mutation and lack of homozygous 243-Met resistant mutant plants in the field.

## Implications for Dinitroaniline Resistance Management

**FIGURE 4:**
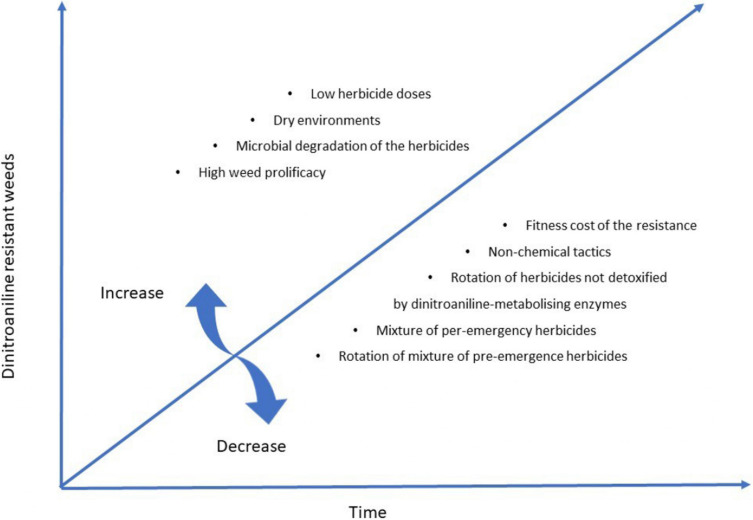
Parameters associated with dinitroaniline resistance evolution in weeds.

In terms of resistance management, there are several implications from dinitroaniline-resistance mechanism research. First, given the recessive nature of TSR, dinitroaniline herbicides should be applied at the higher end of the labeled rates to ensure mortality of plants heterozygous for target-site resistance mutations. Secondly, in the case of NTSR, care should be taken when mixing or rotating herbicides to minimize cross-resistance. It would be wise to rotate dinitroaniline herbicides like trifluralin with herbicides that might not be readily metabolized by dinitroaniline-metabolizing enzymes (e.g., prosulfocarb, pyroxasulfone) (Busi et al., 2017, 2020a). Computer simulation modeling, as well as the screening work with many field *L. rigidum* populations showed that mixtures of pre-emergency herbicides (e.g., trifluralin/prosulfocarb, trifluralin/pyroxasulfone, trifluralin/triallate) can delay the onset of resistance and mitigate the existing levels of resistance (Busi and Beckie, 2020; Busi et al., 2020b). Third, co-existence of TSR and NTSR in the same populations suggest the importance of the integrated weed management (IWM) incorporating non-chemical weed management tactics (harvest weed seed control, crop rotation, etc.) to mitigate resistance evolution, and deployment of competitive crop cultivars to suppress dinitroaniline resistant weeds, especially of mutations with concomitant fitness costs. The main message for dinitroaniline herbicide resistance evolution and management is highlighted in Figure 4.

## Author Contributions

All authors listed have made a substantial, direct and intellectual contribution to the work, and approved it for publication.

## Conflict of Interest

CS is employed by the company Nufarm Limited. The remaining authors declare that the research was conducted in the absence of any commercial or financial relationships that could be construed as a potential conflict of interest.
